# Analysis of washed microbiota transplant efficacy for autism and donor-recipient gut microbiota characteristics

**DOI:** 10.3389/fcimb.2026.1823988

**Published:** 2026-07-01

**Authors:** Yongxi Quan, Mingming Chen, Yao Cai, Li Ling, Yedong Lao, Xingxiang He, Lihao Wu

**Affiliations:** Department of Gastroenterology, The First Affiliated Hospital of Guangdong Pharmaceutical University, Guangzhou, China

**Keywords:** autism, donor-recipient, enterotype, gut microbiota characteristics, washed microbiota transplantation

## Abstract

Emerging evidence suggests that washed microbiota transplantation (WMT) can be used to effectively treat autism spectrum disorder (ASD). This study aimed to evaluate the efficacy of WMT, investigate structural changes in the gut microbiota of children with ASD after WMT, and determine the relationship between donor and recipient gut microbiota characteristics in ASD with different treatment efficacies. This study included 38 ASD patients who underwent a full course of WMT from a single donor between July 2019 and December 2024. The clinical rating scale and stool samples were collected before and 4 weeks after WMT. Using 16S rRNA sequencing, patients were stratified by enterotype, *Bacteroides* (ET-B), or *Prevotella* (ET-P), to compare gut microbiota features with healthy donors and assess how microbial characteristics influence WMT efficacy. Finally, we found that WMT has statistically significant effects on the scale scores changes of core symptoms, sleep disturbances, and gastrointestinal symptoms in patients with ASD. In addition, the efficacy of WMT for ASD was correlated with donor and recipient gut microbiota characteristics before WMT. Among donors and recipients with *Bacteroides* as the dominant genus, the closer the recipient gut microbiota was to that of the donor, the better the efficacy of WMT in patients with ASD.

**Clinical trial registration:**

https://www.chictr.org.cn/index.html, identifier ChiCTR2400089296

## 1 Introduction

Autism spectrum disorder (ASD), including autism, Asperger syndrome, and unspecified generalized developmental disorders, is a neurodevelopmental disorder characterized by social communication disorders, repetitive stereotyped behaviors, and restricted interests (Sharma et al., 2018). In addition to neurobehavioral symptoms, children with ASD frequently exhibit gastrointestinal symptoms and sleep disturbances.

However, the precise etiological mechanisms underlying ASD remain unclear. Emerging evidence indicates a multifactorial pathogenesis involving genetic predisposition, environmental exposures, and epigenetic modifications (de la Torre-Ubieta et al., 2016; Mandy and Lai, 2016; Lee et al., 2019). The absence of definitive biomarkers necessitates reliance on behavioral assessments using gold-standard instruments, including Developmental, Dimensional, and Diagnostic Interview (3di), Childhood Autism Rating Scale (CARS), and Autism Spectrum Disorder Observation for Children (ASD-OC) for diagnostic confirmation (Hirota and King, 2023). Widely implemented clinical tools for severity assessment include the Childhood Autism Rating Scale (CARS) and Autism Behavior Checklist (ABC).

Regarding the relationship between the gut microbiota and ASD, animal studies have shown that colonization of germ-free mice with gut microbiota from donors with ASD can induce characteristic ASD-like behaviors. Repeated transplantation of fecal extracts from children with ASD into pregnant mice can establish an ASD mouse model (Qi et al., 2021). Studies on ASD-related genes have shown that *EPHB6* deficiency can induce gut microbiota imbalance, resulting in vitamin B6 and dopamine deficiency, which in turn induces an excitatory/inhibitory imbalance in prefrontal cortical pyramidal neurons via the dopamine D1 receptor pathway, thereby mediating ASD-like behaviors in mice (Li et al., 2020). Gastrointestinal symptoms are common in patients with ASD and include significant disturbances in gut microbiota metabolism (Xie et al., 2022). Recent studies have confirmed differences in the gut microbiota composition between children with ASD and normally developing children (Li et al., 2023). A meta-analysis (Xu et al., 2019) indicated significant differences in the abundance of gut microbiota (*Akkermansia*, *Bifidobacterium*, *Bacteroides*, *Escherichia coli*, and *Lactobacillus*) between children with ASD and normally developing children. This suggests that dysbiosis or an imbalance of the gut microbiota may be one pathogenic mechanism underlying ASD pathophysiology.

The bidirectional exchange of information between the gut microbiota and the central nervous system is termed “gut-brain axis” (Socała et al., 2021). The gut microbiota plays a central role in the maturation and development of the immune, nervous, and gastrointestinal systems and is also involved in important metabolic pathways (Li et al., 2020). Animal studies indicate that *EPHB6* deficiency induces ASD-like behaviors in mice by triggering gut microbiota dysbiosis, leading to vitamin B6 and dopamine deficiencies (Sorboni et al., 2022). This, in turn, disrupts the excitatory/inhibitory balance in prefrontal cortical pyramidal neurons via the dopamine D1 receptor pathway. A meta-analysis revealed significant differences in gut microbiota composition between children with ASD and typically developing children (Xu et al., 2019). This suggests that dysbiosis or disruption of the gut microbiota may represent a pathogenic mechanism underlying ASD. The gut-brain axis plays a crucial role in ASD and is considered a therapeutic target for ASD.

Fecal microbiota transplantation (FMT) can alleviate ASD-related symptoms by regulating the gut microbiota and metabolism (Li et al., 2022). Washed microbiota transplantation (WMT) is a novel technique based on traditional FMT that uses an intelligent fecal microbiota separation system and rigorous washing processes. Thus, WMT may be an effective, feasible, and safe approach for treating ASD. However, some patients with ASD exhibit suboptimal therapeutic outcomes. We hypothesized that the efficacy of WMT for ASD correlates with alterations in the gut microbiota of patients with ASD and donors. Improving donor–recipient matching may be key to enhancing the efficacy of WMT for ASD. Thus, this study examined the relationship between WMT efficacy in ASD and donor–recipient gut microbiota characteristics.

## 2 Materials and methods

### 2.1 Study participants

This study examined 38 children with ASD who received one course of WMT at our hospital between July 1, 2019, and December 31, 2024. Bacterial suspensions were sourced exclusively from one donor throughout the treatment period, and 19 corresponding donor samples were included. Clinical data and fecal specimens were collected before and four weeks after treatment.

#### 2.1.1 Eligibility criteria

- Children diagnosed with ASD met the criteria outlined in the Diagnostic and Statistical Manual of Mental Disorders, Fifth Edition (DSM-5). These criteria included the following: persistent deficits in social communication and social interaction; restricted, repetitive patterns of behavior, interests, or sensory activities; symptoms onset during early childhood; symptoms resulting in clinically significant impairment in social, occupational, or other major areas of functioning; and symptoms that could not be better explained by intellectual disability or pervasive developmental disorders.
- The child and family were able to cooperate with the collection of comprehensive medical records.
- The subject or the subject’s legal guardian voluntarily participated in this study.

#### 2.1.2 Exclusion criteria

- The patient had taken antibiotics within one month before WMT treatment.
- The patient was diagnosed with other psychiatric disorders, such as schizophrenia or depression, with onset during childhood.
- Patients diagnosed with severe organic brain lesions or other serious heart, lung, liver, or kidney diseases.
- Subjects who experienced severe adverse reactions during FMT administration, including severe diarrhea, persistent abdominal pain, fever, and serious infections.
- Subjects or their legal guardians who wished to withdraw from the study.

### 2.2 Selection of donor

Donors eligible for enrollment were required to meet both the national standards for blood donor health examinations (GB 18467-2011) and the screening criteria for fecal microbiota transplantation donors. First, prospective donors were required to sign an informed consent form for intestinal microbiota donation and complete an online or offline questionnaire to exclude relevant risk factors, such as medication use, personal and family medical history, or high-risk behaviors. If prospective donors met the initial screening standards, they underwent an interview, and a physician made the final decision regarding donor eligibility. Candidates who passed the above screening underwent laboratory testing to exclude infectious diseases and potential microbiota-related dysbiosis. Candidates who passed the above screenings were considered qualified donors. Additional screening tests were conducted before donor enrollment, and donors also completed a questionnaire on the day of donation.

### 2.3 Preparation of washed microbiota suspension and WMT procedure

#### 2.3.1 Preparation of washed microbiota suspension

Donors collected stool samples in a dedicated sampling room in the Microbial Wash Preparation Laboratory at our hospital. A single collection yielded more than 50 g of stool, which was placed in a sterile sampling bucket and processed using an automated fecal-microbial separation system. Using this system, sterile 0.9% sodium chloride solution was added at a mass-to-volume ratio of 1:5 relative to the fecal sample for filtration. The liquid was automatically separated into five sterile centrifuge tubes. The fifth separated solution was centrifuged at 2, 500–5, 000 rpm for three minutes. After removal of the supernatant, fecal bacterial pellets were obtained. A sterile 0.9% sodium chloride solution was added, and the pellet was centrifuged three additional times. The pellet obtained after repeating this process three times constituted the washed microbial community. A sterile 0.9% sodium chloride solution was added at a 1:2 volume ratio and mixed thoroughly to obtain a washed bacterial suspension. The supernatant was retained, and the fecal bacterial suspension was collected. The entire process, from the sample preparation to infusion of the bacterial suspension into the intestine of a child with ASD, was completed within one hour.

#### 2.3.2 WMT procedure

For this study, all patients with ASD underwent transendoscopic enteral tubing (TET) via the lower gastrointestinal tract. On the evening before tube placement, at 8:00 p.m., patients received an oral dose of 20% mannitol (0.5 g/kg). On the morning of tube placement, between 5:00 and 6:00 a.m., patients received an additional oral dose of mannitol, and cleansing enemas were performed as needed until bowel preparation standards were met. Following intubation, the patient was placed in the right lateral decubitus position. The microbial suspension was infused slowly at a rate of 50 mL every 1–2 min. After infusion, the child remained in the right lateral decubitus position for 30 min before being transitioned to the supine position for at least 2 h. The volume of bacterial suspension administered per infusion depended on the child’s age: 30–50 mL for younger children and 90 mL for children older than 7 years. Each treatment course consisted of six consecutive days. Children with ASD enrolled in the study underwent follow-up 4 weeks after completing the first WMT treatment course.

### 2.4 Data collection

#### 2.4.1 General information

General information on enrolled children with ASD was collected from inpatient medical records, including sex, age at initial admission, height, and weight.

#### 2.4.2 Clinical efficacy outcomes

Currently, there are no definitive laboratory indicators that reflect the severity of ASD symptoms or disease progression in children. In clinical practice, the ABC and CARS are commonly used to assess core ASD symptoms (see Appendices 3–6 for details). In this study, clinical effectiveness was defined as follows: patients with any decrease from baseline in total CARS or ABC score (decrease > 0) were assigned to the effective group, while those with no decrease (decrease ≤ 0) were assigned to the ineffective group. Given that this definition is relatively liberal, the findings of this study should be considered exploratory. All scales were administered to children with ASD before they received WMT. We reassessed the data 4 weeks after the first or second WMT treatment cycle. This allowed comparison of the results before and after the intervention. We also examined the relationship between these assessment results and the gut microbiota profiles of children with ASD before WMT.

#### 2.4.3 Fecal samples

Fecal specimens were collected from children with ASD before the first and second courses of single-donor WMT and stored in sterile tubes. Corresponding bacterial suspension samples were collected from the respective donors. All collected samples were stored at -80 °C until shipment to Shanghai Meiji Biotechnology Co., Ltd. for microbiome sequencing.

### 2.5 Statistical analysis

#### 2.5.1 General data analysis

Demographic data of the study participants were analyzed using SPSS version 26.0. Clinical efficacy outcome data were analyzed using SPSS version 26.0 and GraphPad Prism version 9. All clinical efficacy outcome data were tested for normality. Measurement data conforming to a normal distribution are expressed as mean ± standard deviation, and paired-samples t-tests were used for comparisons before and after WMT. Measurement data not conforming to a normal distribution are expressed as median (interquartile range [IQR]), and the Wilcoxon signed-rank test was used for comparisons before and after WMT. Differences were considered statistically significant at P<0.05.

#### 2.5.2 16S rRNA sequencing data analysis

Alpha diversity analysis was used to evaluate the richness and diversity of gut microbiota communities in clinical samples using multiple diversity indices. For example, the Shannon index primarily reflects community diversity, with higher values indicating greater diversity. The Microbial Dysbiosis Index (MDI) measures the degree of microbial ecological imbalance, with higher values indicating greater dysbiosis. We conducted a correlation heatmap analysis between clinical factors [ABC, CARS, and Sleep Disturbance Scale for Children (SDSC) scores] and the top 50 taxa with the highest relative abundance in the gut microbiota of children with ASD before WMT to explore associations between species and clinical factors. At the genus level, species composition and relative abundance were statistically compared among children with ASD with different gut types and treatment efficacies before WMT, as well as among donors. These comparisons were visualized using community bar charts.

## 3 Results

### 3.1 Demographic data

The final sample size included 38 children with ASD after application of the inclusion and exclusion criteria. The sample included 31 males (81.6%) and 7 females (18.4%), with a mean age of 6.9 years (range, 2–15 years). The mean body mass index (BMI) was 15.48 kg/m², ranging from 12.29 to 21.27 kg/m². These demographic characteristics are summarized in **Table 1**.

**Table 1:** Baseline demographic characteristics.

Demographic characteristics	n=38
Male	31 (81.58%)
Female	7 (18.42%)
Age (year)	6.9 (2 -15)
BMI (kg/m2)	15.48 (12.29 - 21.27) (n=37)

### 3.2 Clinical efficacy analysis of WMT in ASD

#### 3.2.1 Effects of WMT on core ASD symptoms, sleep, and gastrointestinal symptoms in children with ASD

**Figure 1:**
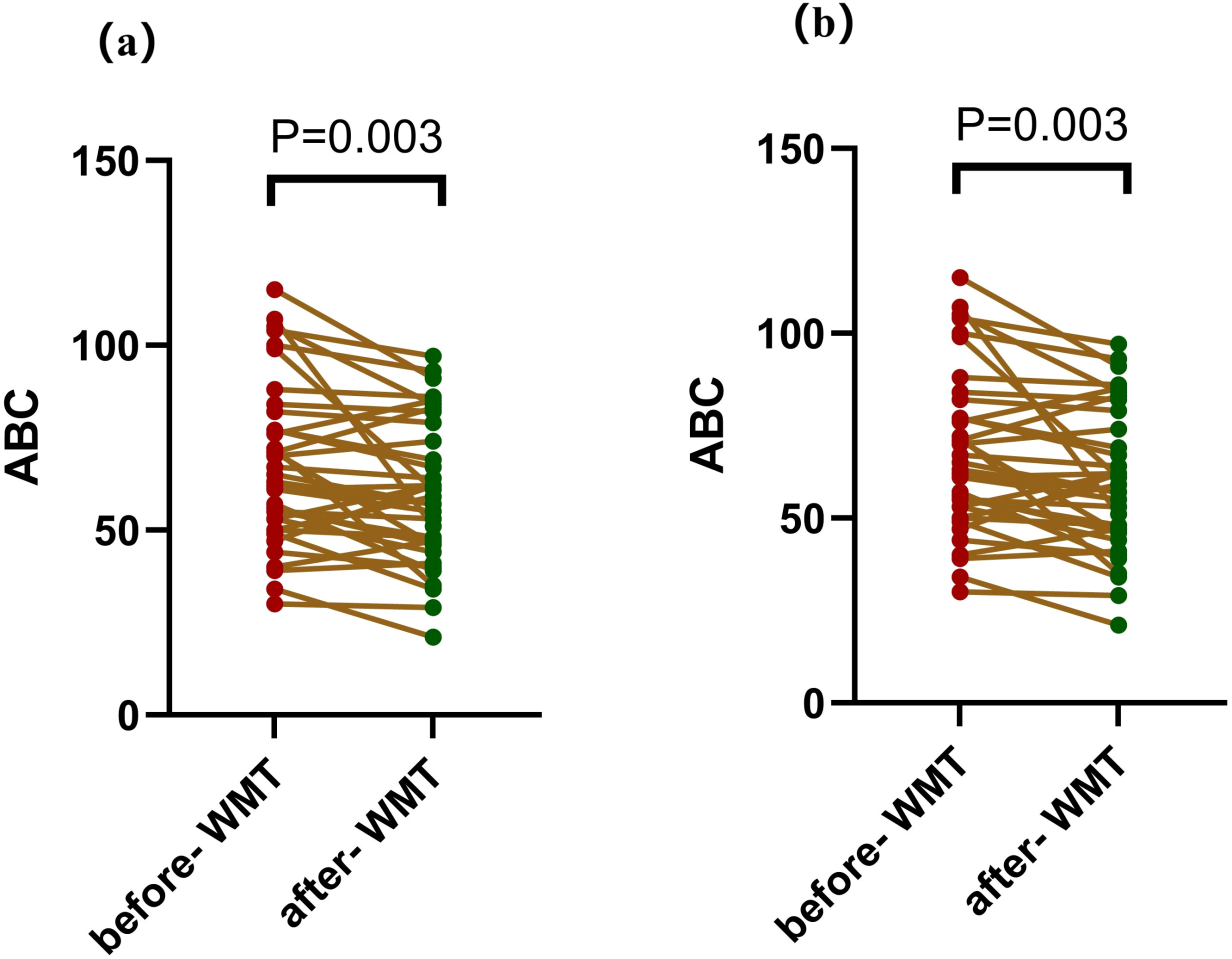
Comparison of changes in ABC and CARS scale scores in children with ASD before and after single-donor WMT. **(a)** comparison of ABC scale scores; **(b)** comparison of CARS scale scores.

**Figure 2:**
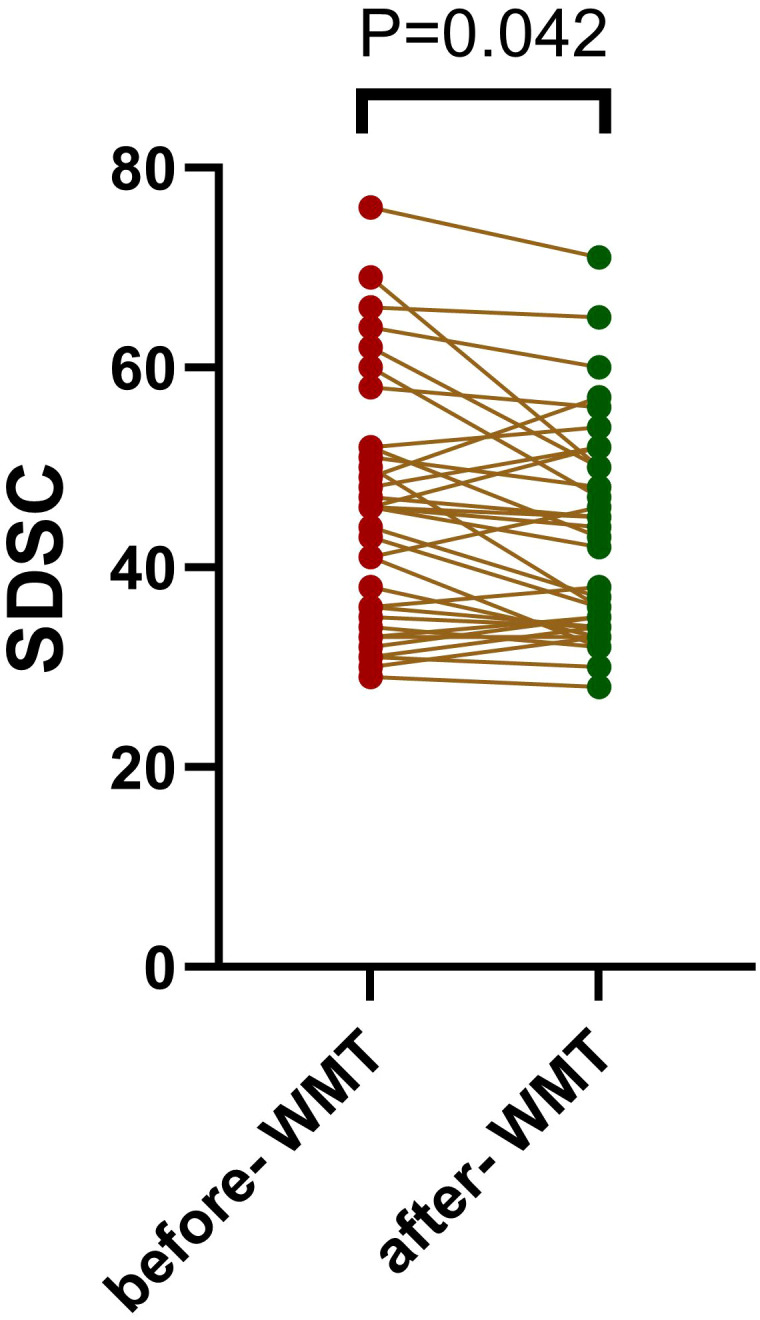
Comparison of changes in SDSC scale scores in children with ASD before and after single-donor WMT.

**Figure 3:**
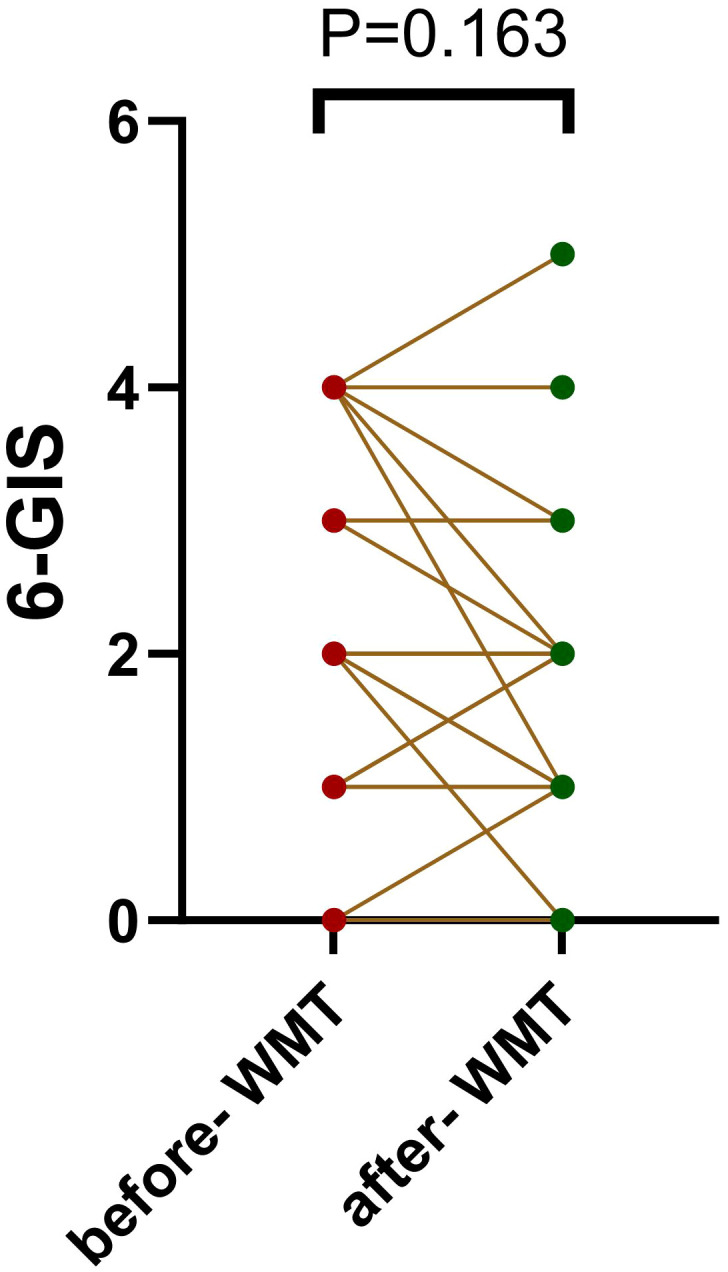
Comparison of changes in 6-GIS scale scores in children with ASD before and after single-donor WMT.

The present study included 38 children diagnosed with ASD, of whom 37 underwent ABC scale assessment before and after the first course of single-donor WMT. This analysis examined ABC scores in children with ASD before and after WMT. The results presented in **Table 2**, **Figure 1A** demonstrated a significant decrease in ABC scores. This decrease was statistically significant (*P* = 0.003). A total of 38 children with ASD were assessed using the CARS. The results presented in **Table 2**, **Figure 1B** demonstrated a significant decrease in CARS scores. CARS scores decreased from 36.00 (33.38, 37.50) to 33.75 (30.88, 36.50), with statistical significance at *P* < 0.0001. A total of 34 children underwent assessment using the SDSC. The results (**Table 2**; **Figure 2**) demonstrated that after WMT, children with ASD exhibited a statistically significant decrease in SDSC scores [46.00 (34.75, 52.00) vs. 42.50 (34.00, 50.50), *P* = 0.042]. Furthermore, 23 children underwent assessment using the six-item Gastrointestinal Severity Index (6-GIS). The results presented in **Table 2**, **Figure 3** revealed no statistically significant difference in 6-GIS scores after WMT compared with those before WMT (2.00 [1.00, 3.00] vs. 2.00 [1.00, 2.00], *P* = 0.163). The median and lower quartiles of 6-GIS scores in children with ASD remained unchanged before and after WMT, while the upper quartile decreased after treatment.

**Table 2:** Effects of single-donor WMT on ABC, CARS, SDSC and 6-GIS scale scores in pediatric ASD patients.

Rating Scale	Before- WMT	After- WMT	P
ABC	67.27 ± 21.7 (n=37)	59.68 ± 19.22 (n=37)	0.003
CARS	36.00 (33.38, 37.50) (n=38)	33.75 (30.88, 36.5) (n=38)	< 0.0001
SDSC	46.00 (34.75, 52.00) (n=34)	42.50 (34.00, 50.50)(n=34)	0.042
6-GIS	2.00 (1.00, 3.00) (n=23)	2.00 (1.00, 2.00) (n=23)	0.163

### 3.3 Analysis of gut microbiota community composition

#### 3.3.1 Analysis of gut microbiota characteristics in children with ASD with different gut types following WMT

**Figure 4:**
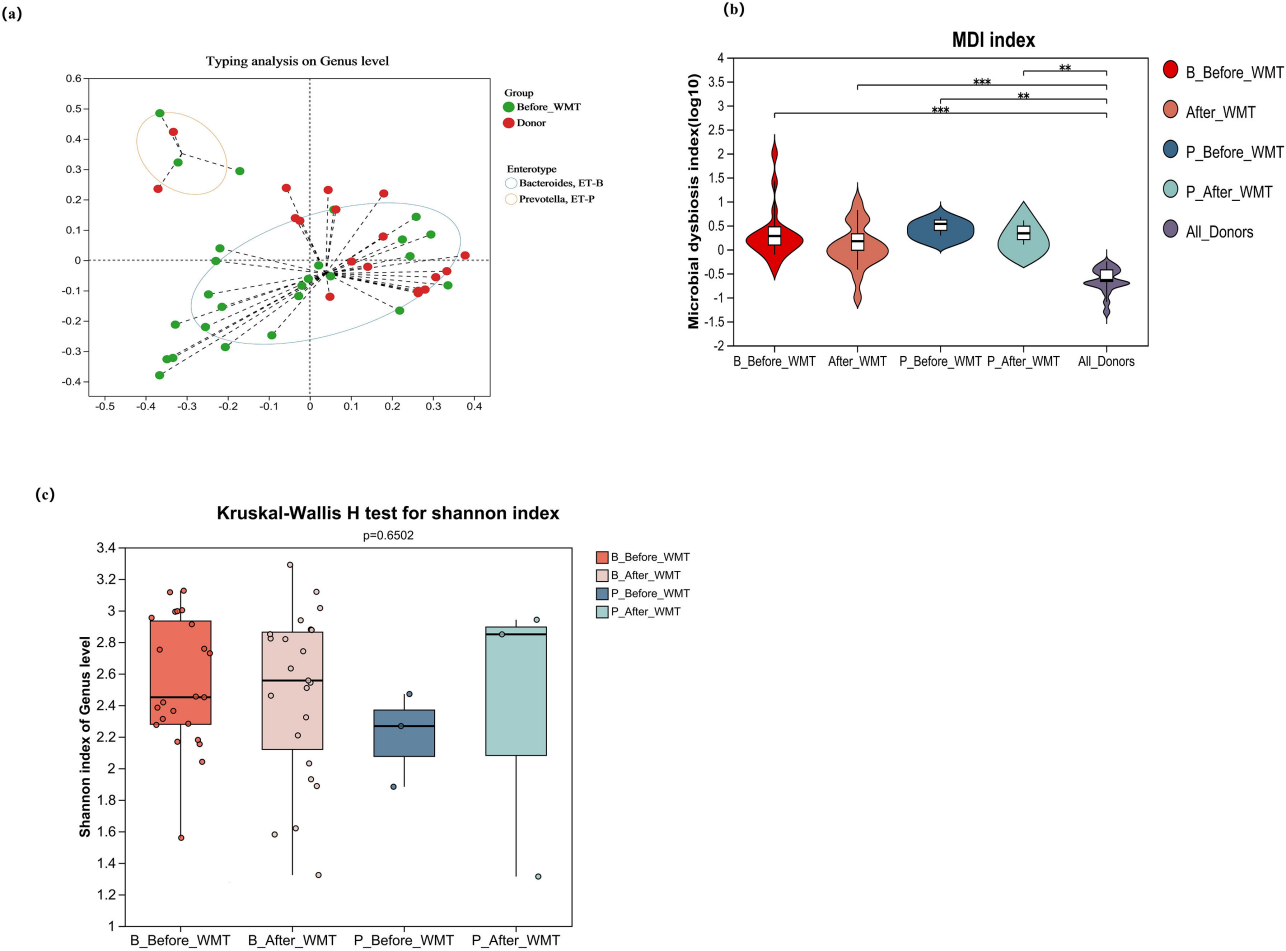
Analysis of gut microbiota characteristics in ASD children and donors before and after WMT based on two Q22 intestinal types. **(a)** Genus-level classification analysis of ASD children and donor gut microbiota before WMT; **(b)** Dysbiosis index analysis at the genus level for ASD patients with two intestinal types before and after WMT, and healthy donors (Wilcoxon signed-rank test); **(c)** Alpha diversity (Shannon index) analysis for ASD patients with two intestinal types before and after WMT (Kruskal-Wallis H test). P < 0.05 indicates statistical significance; one asterisk denotes P < 0.05, two asterisks denotes P < 0.01, three asterisks denotes P < 0.001.

Gut microbiota profiles were analyzed based on the dominant bacterial communities in fecal samples collected from children with ASD before WMT and from healthy donors. The results revealed that both children with ASD before WMT and healthy donors exhibited two distinct gut microbiota patterns: a *Bacteroide*s-dominant pattern (designated ET-B) and a *Prevotella*-dominant pattern (designated ET-P). The microbiota was predominantly composed of *Bacteroides* and *Prevotella*. In the cohort of patients with ASD, 23 (88.46%) exhibited ET-B, whereas 3 (11.54%) exhibited ET-P before WMT. In contrast, among healthy donors, 17 cases (89.47%) exhibited ET-B, whereas two donors (10.53%) exhibited ET-P (**Figure 4a**).

Baseline analysis relative to the healthy donor group revealed changes in the MDI before and after WMT in ASD groups with different gut types. The ET-B and ET-P groups demonstrated significantly elevated MDI values before and after WMT compared with the healthy donor group. Differences were considered statistically significant at *P* < 0.05. After WMT, the MDI declined in both the ET-B and ET-P groups. However, the ET-P group demonstrated higher MDI values than the ET-B group, both before and after WMT. These findings suggest gut microbiota dysbiosis in children with ASD. The ET-P group demonstrated more severe dysbiosis than the ET-B group (**Figure 4b**).

Compared with before WMT levels, both ET-B and ET-P groups exhibited elevated Shannon indices after WMT. However, the intergroup difference was not statistically significant (*P* = 0.6502). Comparison of the ET-P and ET-B groups revealed that the ET-P group exhibited a lower Shannon index before WMT, whereas the ET-B group exhibited a higher Shannon index after WMT (**Figure 4c**).

#### 3.3.2 Relationship between WMT efficacy and gut microbiota characteristics in donor–recipient pairs based on gut type analysis

The objective of this analysis was to explore the potential associations between WMT efficacy in ASD and gut microbiota characteristics in donor–recipient pairs. To this end, the gut microbiota profiles were analyzed according to gut type of children with ASD who responded effectively or ineffectively to WMT. After excluding children with ASD missing questionnaire scores, 16 donor–recipient pairs were classified as WMT responders or non-responders.

Donors and recipients were grouped according to gut type as follows: ET-B recipients matched with ET-B donors and ET-B recipients matched with ET-P donors. The ET-B recipient–ET-B donor group was designated as the BB-matched group (n=14), whereas the ET-B recipient–ET-P donor group was designated as the BP-matched group (n=2). Within the BB- and BP-matched groups, patients were further categorized according to WMT efficacy as follows: BB-matched effective group (n = 6), BB-matched ineffective group (n = 8), BP-matched effective group (n = 1), and BP-matched ineffective group (n = 1). WMT efficacy was determined by decreases in ABC, CARS, and SDSC scores after the initial WMT course compared with pretreatment baseline values. WMT treatment failure was defined as the absence of improvement in ABC, CARS, or SDSC scores after the initial WMT course compared with pretreatment levels.

**Figure 5:**
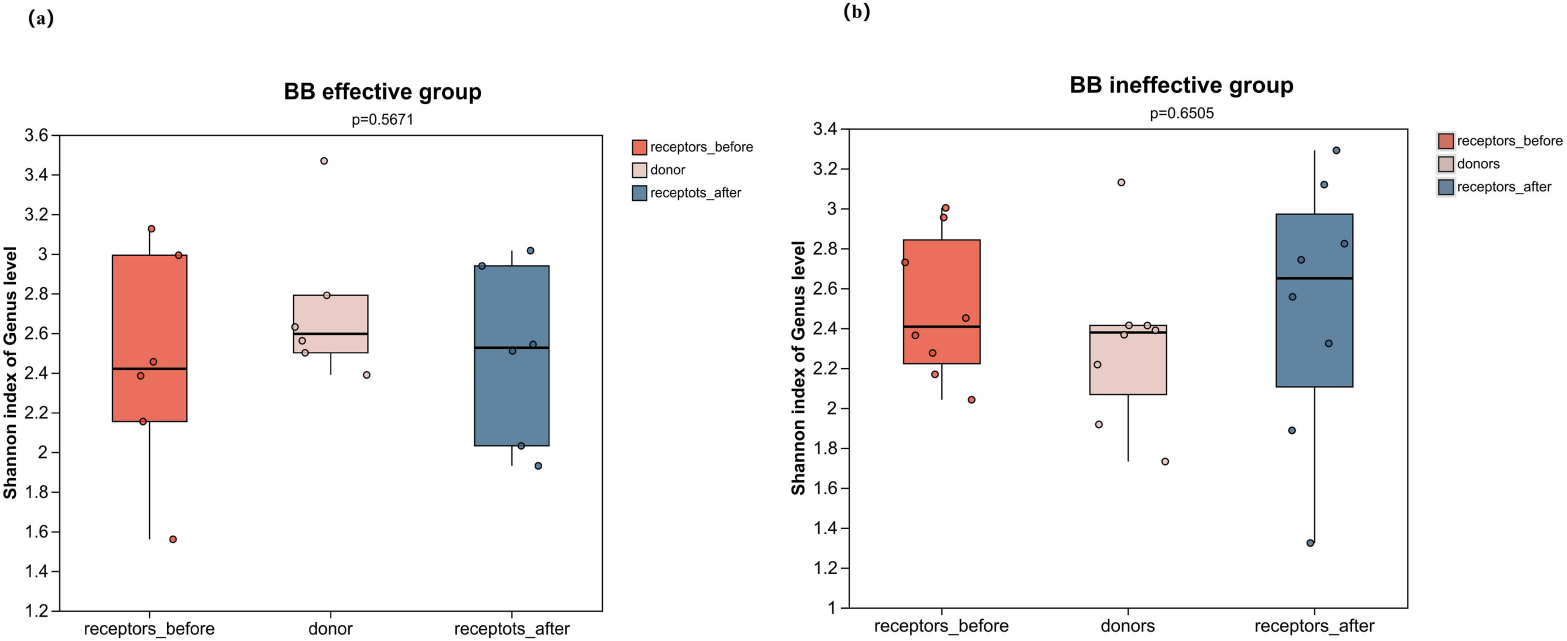
**(a)** Alpha diversity analysis (Shannon index) of recipients in the effective BB-matched group before and after WMT compared with donors; **(b)** Alpha diversity analysis (Shannon index) of recipients in the ineffective BB-matched group before and after WMT compared with donors. Kruskal-Wallis H test was used for all comparisons. P < 0.05 indicates statistical significance.

Owing to the limited sample sizes in the BP-matched effective and ineffective groups, this analysis examined the alpha diversity of the gut microbiota in BB-matched effective and ineffective recipients before and after WMT compared with donors. The results demonstrated that the Shannon index of the gut microbiota in BB-matched effective recipients was lower before WMT, whereas donor Shannon indices increased after WMT, resulting in recipient levels approaching those of donors. In contrast, the Shannon index of the BB-matched ineffective group remained higher than that of the donor group both before and after WMT, with a continued increase after treatment that was not statistically significant (*P*>0.05; **Figures 5a, b**). This finding suggests that in ET-B donor–recipient matching, WMT can promote gut microbiota diversity in children with ASD, irrespective of treatment efficacy.

**Figure 6:**
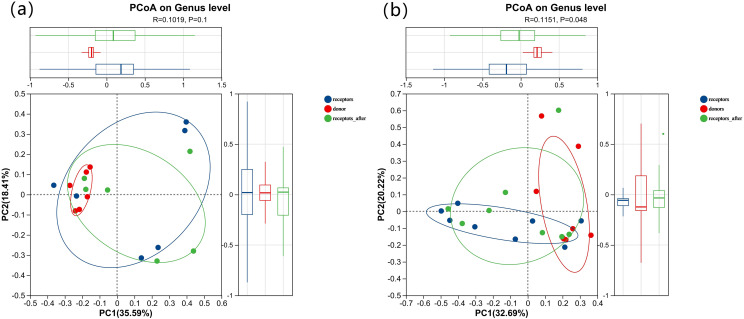
**(a)** PCoA analysis of relative abundance at the genus level of gut microbiota between recipients and donors in the BB-matched effective group; **(b)** PCoA analysis of relative abundance at the genus level of gut microbiota between recipients and donors in the BB-matched ineffective group. ANOSIM tests were performed for both groups, with P < 0.05 indicating statistical significance. All comparisons used the Wilcoxon signed-rank test; P < 0.05 indicates statistical significance.

We further investigated similarities and differences in gut microbiota community composition between recipients and donors before and after WMT treatment in the BP-matched groups using principal coordinates analysis. The results demonstrated that before and after WMT, Bray–Curtis (BC) distances between recipient and donor gut microbiota formed three groups (**Figures 6a, b**). In the effective BB-matched group, no significant differences were observed in BC distances between recipients and donors before and after WMT (**Figure 6a**, *R* = 0.1019, *P* = 0.1). However, compared with pretreatment levels, recipient gut microbiota BC distances after WMT converged toward donor levels, indicating that the gut microbiota community composition in WMT-treated recipients of the effective BB-matched group approached donor profiles. In the ineffective BB-matched group, significant differences were identified in BC distances between recipient and donor gut microbiota before and after WMT (**Figure 6b**, *R* = 0.1551, *P* = 0.048). This finding suggests that in the ineffective BB-matched group, substantial differences existed between recipient and donor gut microbiota community composition before WMT, and these differences persisted after WMT. This finding indicated that in the BB-matched group, the effective group exhibited greater similarity in gut microbiota community composition to donor profiles before WMT and greater convergence toward donor profiles after WMT.

**Figure 7:**
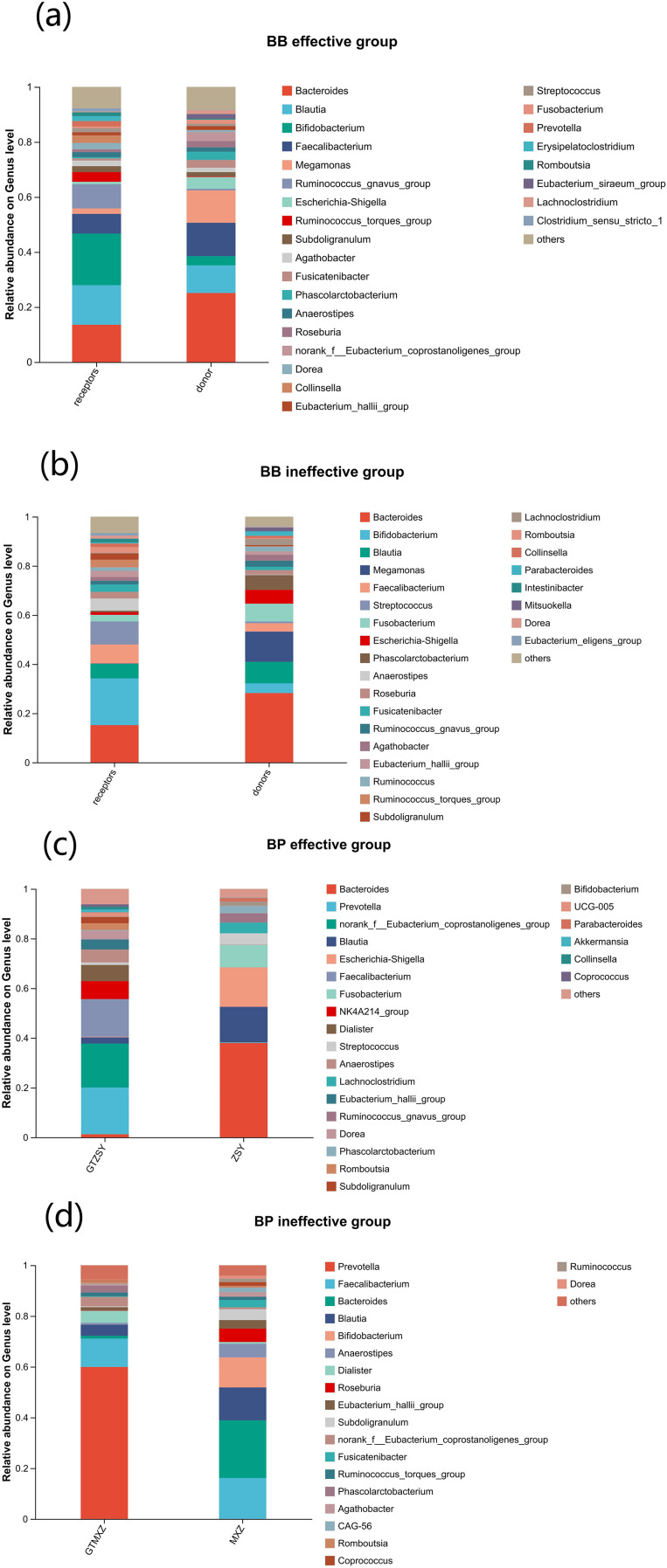
Bar chart showing gut microbiota community composition analysis at the genus level between donor and recipient groups: **(a)** BB matched effective group; **(b)** BB matched ineffective group; **(c)** BP matched effective group; **(d)** BP matched ineffective group. All comparisons were performed using the Wilcoxon signed-rank test. P < 0.05 indicates statistical significance.

A comparative analysis was conducted on the relative abundance of dominant genera in recipient gut microbiota before WMT relative to donor microbiota across the BB-matched effective, BB-matched ineffective, BP-matched effective, and BP-matched ineffective groups (**Figures 7a–d**). (1) BB-matched effective group: the five most abundant genera in donors were *Bacteroides, Blautia, Faecalibacterium, Escherichia-Shigella*, and *Bifidobacterium*; the five most abundant genera in recipients were *Bifidobacterium, Blautia, Bacteroides, Ruminococcus_gnavus_group*, and *Faecalibacterium*. (2) BB-matched ineffective group: the five most abundant genera in donors were *Bacteroides, Megamonas, Blautia, Fusobacterium*, and *Phascolarctobacterium*; the five most abundant genera in recipients were *Bifidobacterium, Bacteroides, Streptococcus, Faecalibacterium*, and *Blautia*. (3) BP-matched effective group: the five most abundant genera in donors were *Prevotella, norank_f_*_*Eubacterium_coprostanoligenes_group, Faecalibacterium, Bacteroides*, and *Dialister*; the five most abundant genera in recipients were *Bacteroides, Escherichia-Shigella, Blautia, Fusobacterium*, and *Streptococcus*. (4) BP-matched ineffective group: the five most abundant genera in donors were *Prevotella, Faecalibacterium, Dialister, Blautia*, and *norank_f:Eubacterium_coprostanoligenes_group*; the five most abundant genera in recipients were *Bacteroides, Faecalibacterium, Blautia, Bifidobacterium*, and *Anaerostipes*.

**Figure 8:**
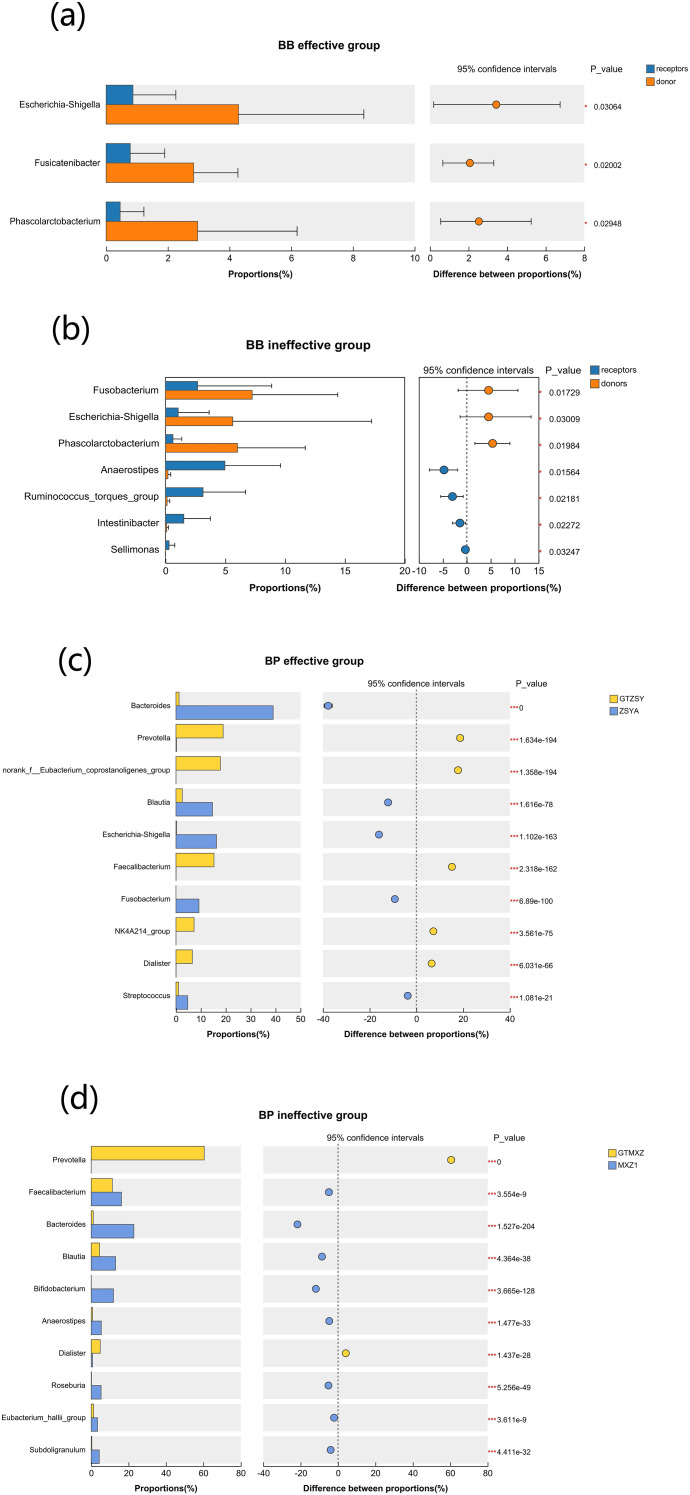
Analysis of species differences in gut microbiota between donor and recipient at the genus level: **(a)** BB matched effective group; **(b)** BB matched ineffective group; **(c)** BP matched effective group; **(d)** BP matched ineffective group. **(a, b)** employed the Wilcoxon signed-rank test; **(c, d)** employed the chi-square test. P < 0.05 indicates statistical significance.

We compared gut microbiota composition between recipients and donors prior to WMT across BB-matched effective, BB-matched ineffective, BP-matched effective, and BP-matched ineffective groups. The analysis revealed significant differences in the gut microbiota composition between donors and recipients at the genus level (*P* < 0.05). The results are shown in **Figures 8a–d**. (1) BB-matched effective group: compared with donors, recipients exhibited significantly lower relative abundances of *Escherichia-Shigella, Fusobacterium*, and *Phascolarctobacterium* (results shown in **Figure 6**). (2) BB-matched ineffective group: compared with donors, recipients exhibited higher relative abundances of *Anaerostipes, Ruminococcus_gnavus_group, Intestinibacter*, and *Sellimonas*; lower relative abundances of *Fusobacterium*, *Shigella*, and *Vibrio* (results shown in **Figure 6**). (3) BP-matched effective group compared with donors, recipients exhibited higher relative abundances of *Bacteroides, Blautia, Escherichia-Shigella, Fusobacterium*, and *Streptococcus*; lower relative abundances of *Prevotella, norank_f:Eubacterium_coprostanoligenes_group, Faecalibacterium, Dialister*, and *NK4A214_group*. (4) BP-matched ineffective group: compared with donors, recipients exhibited higher relative abundances of *Faecalibacterium, Bacteroides, Blautia, Bifidobacterium, Anaerostipes, Roseburia, Eubacterium_hallii_group*, and *Subdoligranulum* in their gut microbiota; lower relative abundances of *Prevotella* and *Bacillus* were observed.

## 4 Discussion

### 4.1 Clinical efficacy of WMT for ASD

Studies have shown that FMT can improve ASD and related symptoms. In a small open-label clinical study (Kang et al., 2017), 18 children with ASD received FMT after two weeks of antibiotic treatment and bowel cleansing. Significant improvements were observed in both ASD-related and gastrointestinal symptoms. These improvements persisted throughout the 8-week follow-up period. Of these children, eight continued to show improvements in these symptoms two years after receiving FMT (Kang et al., 2019). In another open-label study (Li et al., 2024), 38 children with ASD received capsules containing fecal microbiota from healthy donors for 12 weeks. This resulted in decreased ABC, CARS, SDSC, and GSRS scores compared with baseline values. None of the patients experienced any serious short- or long-term side effects. Other studies from our research center also found that WMT is effective in improving ASD-related symptoms (Pan et al., 2022; Zhang et al., 2022). Additionally, our findings showed that WMT has statistically significant effects on the scale scores changes of core symptoms, sleep disturbances, and gastrointestinal symptoms in patients with ASD.

### 4.2 Analysis of the relationship between efficacy and gut microbiota in donor–recipient pairs based on gut type

Gut microbiota can be classified into three enterotypes based on dominant bacterial taxa and their functional characteristics. Three major enterotypes have been described: *Bacteroides*, *Prevotella*, and *Ruminococcus* (Arumugam et al., 2011). A Korean study examined the relationship between the gut microbiome and clinical characteristics in patients with ASD. The study grouped patients into two gut types, E1 and E2, based on their gut microbiota composition (Jung et al., 2024). Wei et al. analyzed the gut microbiota of 13 children with ASD with different gut types, identifying three enterotypes: *Prevotella*, *Bacteroides*, and mixed enterotypes. Among these enterotypes, the *Prevotella* enterotype exhibited greater microbial diversity than the *Bacteroides* enterotype. In this study, children with ASD were divided into two groups based on gut type. This classification was based on pre-WMT microbiota profiles and donor microbiota. There were two enterotypes: *Bacteroides* (ET-B) and *Prevotella* (ET-P). Among children with ASD, ET-B and ET-P patterns were also observed in donors. Before WMT, the ET-B group exhibited a higher Shannon index (community diversity) than the ET-P group, consistent with the findings of Wei et al. A study analyzing the gut microbiota of children with ASD revealed that, compared with typically developing children, children with ASD exhibited significantly increased abundances of *Bacteroides* at both the phylum and genus levels (Zou et al., 2020). However, a meta-analysis found lower proportions of *Akkermansia, Bacteroides, Bifidobacterium, and Parabacteroides*, and higher proportions of *Faecalibacterium* in children with ASD (Xu et al., 2019). However, the findings regarding *Bacteroides* abundance in children with ASD were contradictory between these two studies. Furthermore, the significantly higher proportion of ET-B than ET-P in children with ASD in this study contradicts findings from our previous domestic research. This reflects the diversity and heterogeneity of the gut microbiota in children with ASD.

A study of FMT in China showed that different donors could exert different effects across diseases (Chen et al., 2020). This suggests that selecting an effective donor based on the gut microbiota characteristics and matching the donor with a suitable recipient may be key to improving the efficacy of FMT for ASD. Vermeire et al. showed that the efficacy of FMT for inflammatory bowel disease depends on the diversity of bacterial species in the donor gut and the degree of bacterial engraftment in the recipient (Vermeire et al., 2016). Our study also found that gut microbiota diversity in children with ASD in the BB-matched group increased and approached donor levels after WMT. The composition of the gut microbiota became more similar to the donor profile after treatment. This suggests that the efficacy of WMT for ASD is related to the degree of donor microbiota engraftment in the recipient gut.

A previous study indicated that the colonization success rate of gut microbiota species was higher for existing species than for newly introduced species. Thus, if a particular species is already present in the recipient, it is more likely to successfully colonize the recipient gut (Li et al., 2016). He et al. examined the efficacy of FMT for inflammatory bowel disease and *Clostridioides difficile* infection in relation to donor–recipient microbiota profiles. Before FMT, patients in the effective and ineffective groups exhibited distinct clustering patterns in gut microbiota distance relative to their respective donors. However, compared with the ineffective group, the effective group demonstrated a shorter BC distance between the post-FMT microbiota and donor microbiota (He et al., 2022). The present study found that BC distances between children with ASD in the ineffective BB-matched group and their donors differed significantly before and after WMT. In contrast, BC distances between children with ASD in the effective BB-matched group and their donors showed no significant differences before and after WMT. However, BC distances between children with ASD and donors after WMT were smaller than those before WMT. We found that the more similar the gut microbiota community structure of children with ASD was to that of donors before WMT, the more effective donor microbiota colonized the recipient gut, resulting in greater therapeutic efficacy of WMT for ASD.

To date, no studies have examined the differences in gut microbiota composition in children with ASD before and after WMT based on donor gut type and treatment efficacy. We found that when patients with ASD with ET-B were matched with ET-B donors, the relative abundances of *Escherichia-Shigella, Fusobacterium*, and *Phascolarctobacterium* were significantly lower in recipients than in donors, which may increase the likelihood of benefiting from WMT. In contrast, higher relative abundances of *Anaerostipes, Ruminococcus_gnavus_group, Intestinibacter*, and *Sellimonas* in recipients, or lower relative abundances of *Fusobacterium, Escherichia-Shigella*, and *Phascolarctobacterium* compared with donors, may be associated with poorer WMT efficacy in children with ASD. When patients with ASD with ET-B matched with ET-P donors, the relative abundances of *Bacteroides, Blautia, Escherichia-Shigella, Fusobacterium*, and *Streptococcus* were significantly higher in recipients than in donors, whereas the relative abundances of *Prevotella, norank_f_Eubacterium_coprostanoligenes_group, Faecalibacterium, Dialister*, and *NK4A214_group* were significantly lower, which may increase the likelihood of benefiting from WMT. Higher relative abundances of *Faecalibacterium, Bacteroides, Blautia, Bifidobacterium, Anaerostipes, Roseburia, Eubacterium_hallii_group*, and *Subdoligranulum* in recipients, along with lower relative abundances of *Prevotella* and *Dialister* compared with donors, may be associated with poorer WMT efficacy in children with ASD.

WMT is a promising and novel approach for treating the core and comorbid symptoms of ASD. This study provides evidence for the efficacy of WMT in addressing both core and comorbid ASD symptoms, while also offering guidance for optimizing donor–recipient matching to enhance therapeutic outcomes in clinical practice. However, this study also has the following limitations: (1) The lack of a blank control group and the small sample size result in insufficient statistical power. Given that this is a small-sample, single-arm study, the observed changes in symptoms and gut microbiota in children with ASD following WMT treatment should be considered preliminary, and these findings require validation in larger, controlled studies. (2) The definition of clinical effectiveness is relatively liberal (any decrease in scale score was classified as effective). Although this definition was based on clinical practical considerations, it may overestimate the treatment effect. Therefore, the conclusions of this study need to be verified in larger, controlled studies. (3) The analysis was limited by a small number of treatment cycles and a short follow-up period (4 weeks), resulting in a superficial analysis of gut microbiota characteristics between effective and ineffective groups of children with ASD and their donors. (4) Additionally, due to constraints, potential confounding factors that may affect symptom changes in ASD, such as dietary habits, lifestyle, or other behavioral interventions, cannot be fully excluded. To address these limitations, we plan to conduct a retrospective study in the future using historical clinical data from our hospital as a natural comparison, which may help partially compensate for the lack of sample size and a control group without immediately expanding the prospective sample size.

## Data Availability

The raw data supporting the conclusions of this article will be made available by the authors, without undue reservation.
