# Supplementary material for: Analysis of washed microbiota transplant efficacy for autism and donor-recipient gut microbiota characteristics
Source: Front Cell Infect Microbiol. 2026 Jul 1;16:1823988. doi: 10.3389/fcimb.2026.1823988 (PMC13368480; doi:10.3389/fcimb.2026.1823988)

Appendices 1. Donor questionnaire


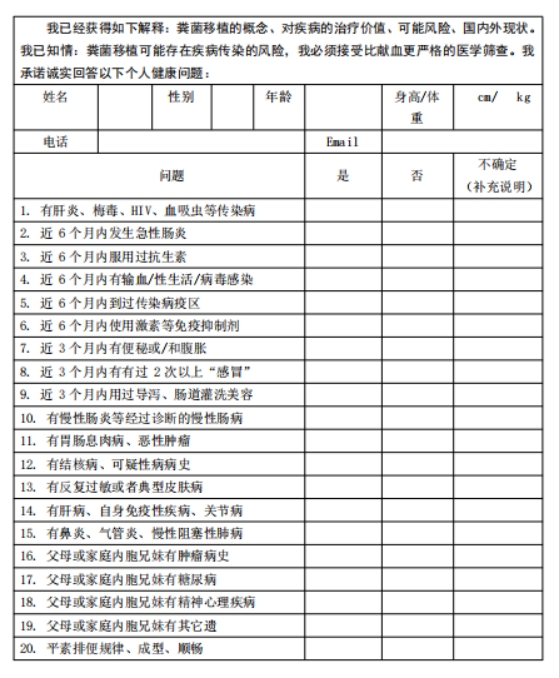


Appendices 2. Donor laboratory screening project list


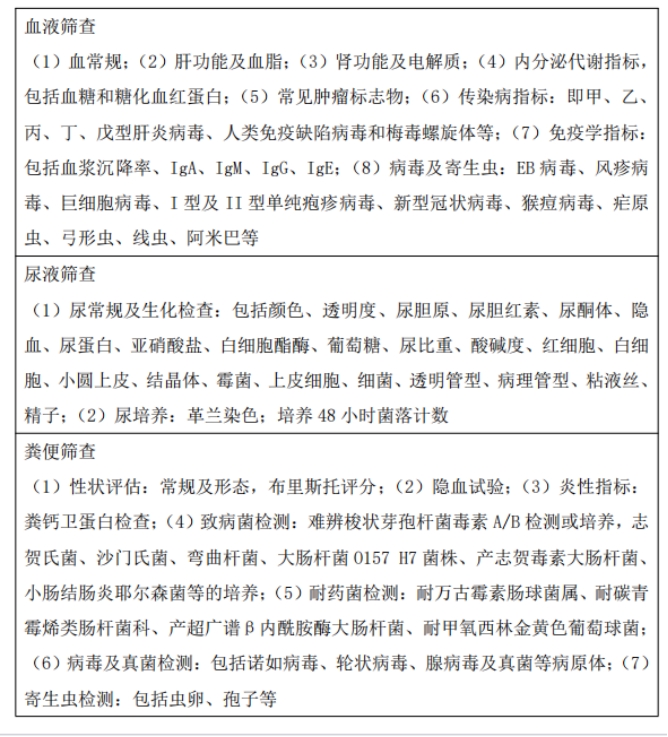


Appendices 3. ABC scales


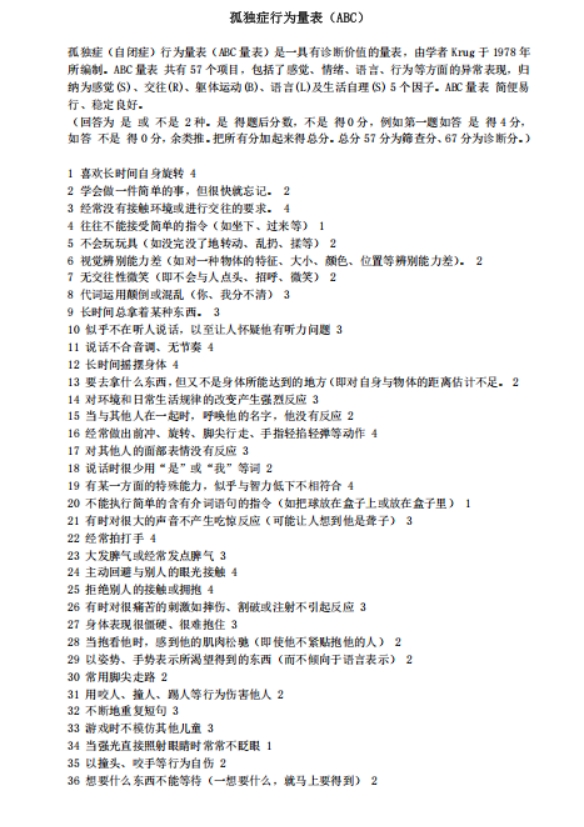


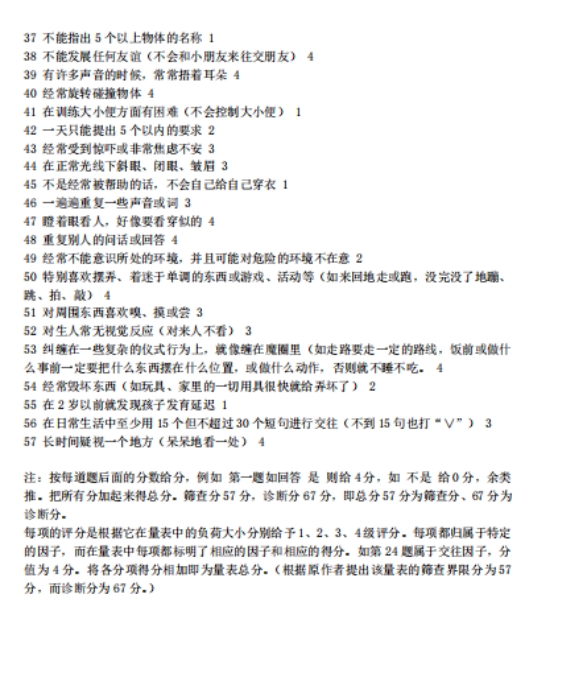


Appendices 4. CARS scales


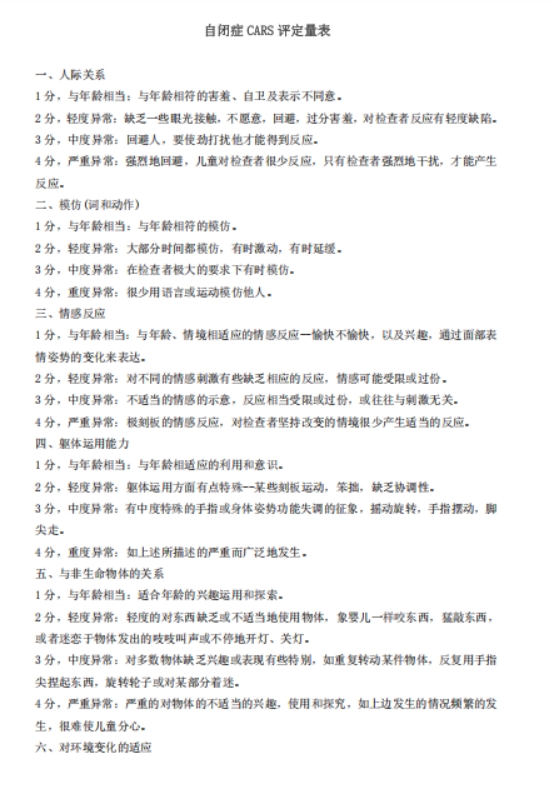

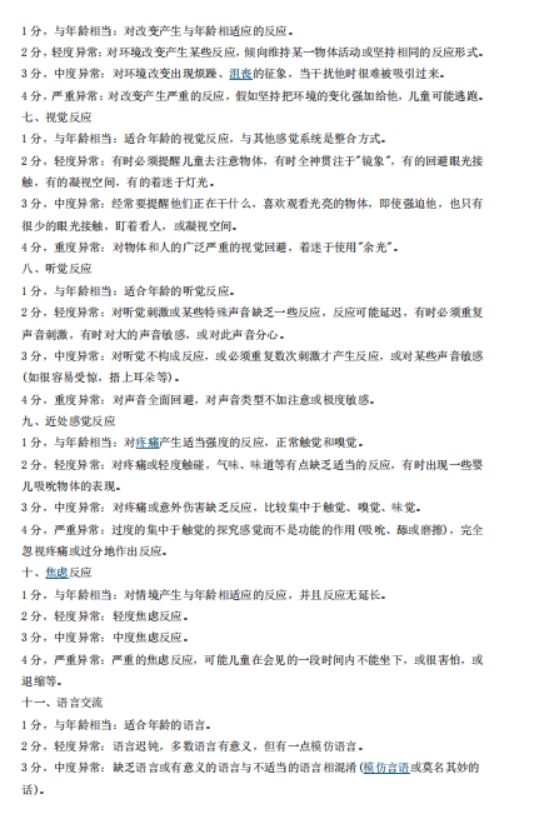

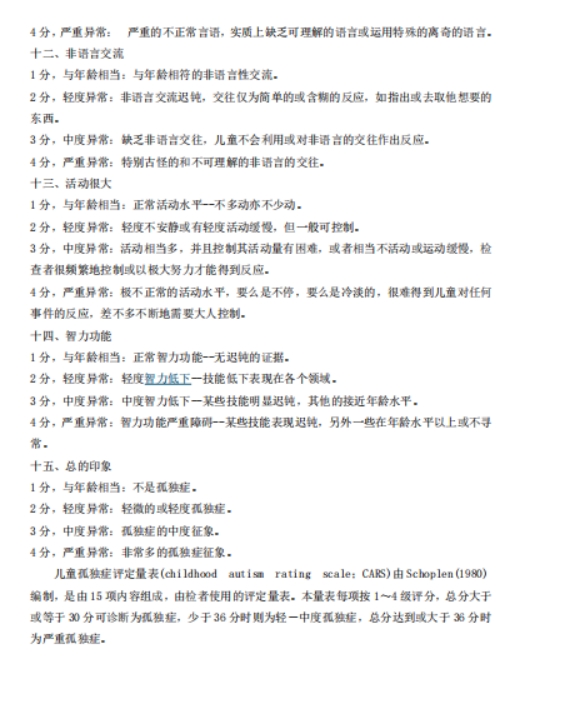


Appendices 5. SDSC scales


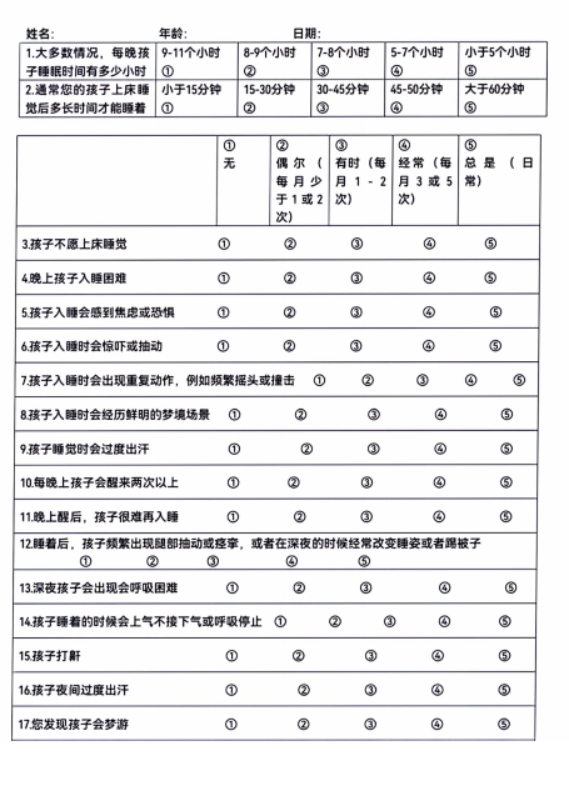


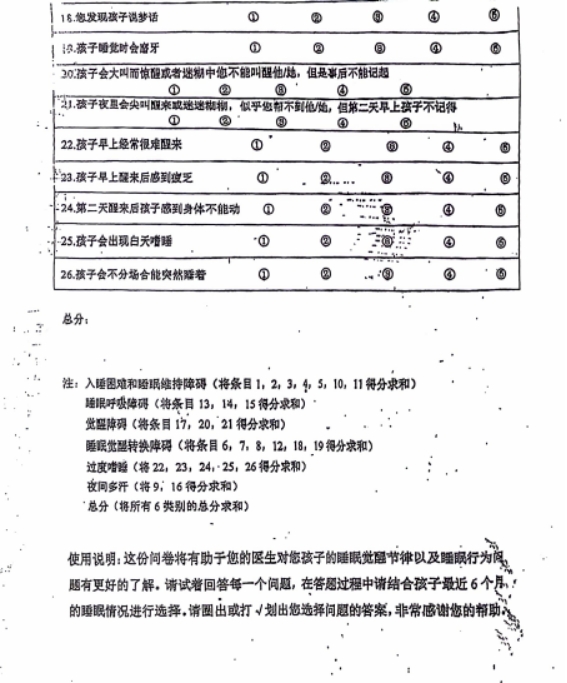


Appendices 6. 6-GIS scales


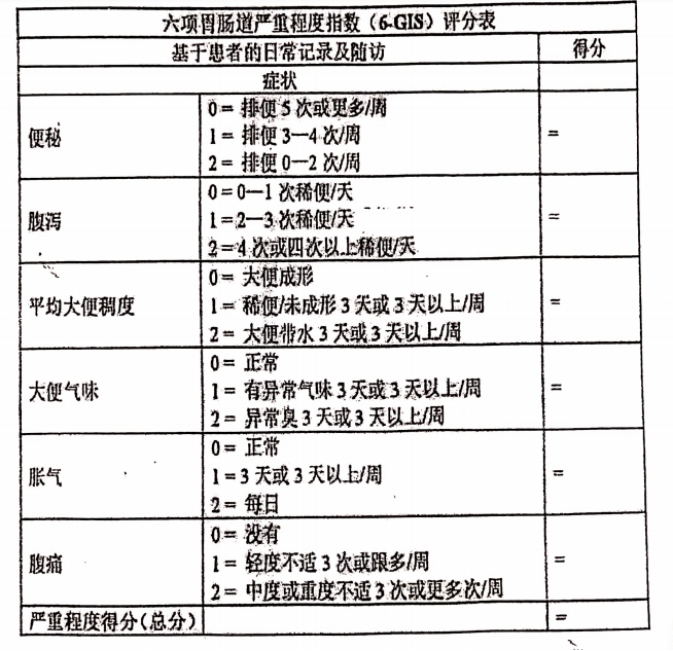

Supplement: Supplementary file 1 [file Table1.docx]
